# Splenosis of the abdomen and pelvis complicated by torsion of a splenic implant clinically mimicking an acute bowel ischemia

**DOI:** 10.1259/bjrcr.20180024

**Published:** 2018-05-24

**Authors:** Alessandro A Lemos, Silvia Crespi, Stefano Costa, Aldo Marini

**Affiliations:** 1 Department of Radiology, Ca’ Granda IRCSS Maggiore Policlinico Hospital Foundation Trust, Milan, Italy; 2 Department of General and Emergency Surgery, Ca’ Granda IRCSS Maggiore Policlinico Hospital Foundation Trust, Milan, Italy

## Abstract

We present a case of splenosis of the abdomen and pelvis complicated by torsion of a splenic implant in a young female patient clinically mimicking an acute bowel ischemia. Splenosis is a benign condition defined as heterotopic auto-transplantation of splenic tissue throughout different body areas. It may occur after rupture of the spleen, either traumatic or secondary to surgical procedures. Although the presence of heterotopic splenic tissue is often asymptomatic and an incidental finding, it may present with sudden abdominal pain and bleeding. CT and MRI play a critical role in the detection of splenosis-related complications, such as torsion of the vascular pedicle and infarction. Splenosis torsion is extremely rare and it is still a diagnostic dilemma; the complication of abdominal splenosis should be considered in the differential diagnosis in patients with previous splenectomy.

## Background

Splenosis is a benign condition defined as heterotopic auto-transplantation of splenic tissue throughout different body areas. It may occur after rupture of the splenic capsule, either from trauma or surgery.^1, 2^ It is estimated that splenosis develop in approximately 70% of splenectomies.^3, 4^ In addition, direct dissemination of splenic tissue to other body compartments may also occur during embryonic development and after laparoscopic splenectomy, because the retrieval port site is a potential source of dissemination.^4^ Therefore, parenchymal disruption must be contained to avoid recurrence.^4^ Splenosis differs from accessory spleens (the so-called splenunculi) and polysplenia because both are congenital conditions unrelated to splenectomy. Splenunculi are usually fewer in number, and in contrast to splenosis, in which the implants are supplied from nearby vessels; splenunculi are supplied by the splenic artery and are usually found near the splenopancreatic or gastrosplenic ligament.^1, 2^ Symptoms are vague and vary according to the location of the splenic tissue. Although the presence of heterotopic splenic tissue is often asymptomatic and an incidental finding, it may present with sudden abdominal pain and bleeding and confused with various acute or chronic disorders.^2, 5,6^k Splenosis is usually an incidental finding at ultrasound as well, which cannot define the splenic origin of the lesions. CT plays a critical role in the diagnosis of splenosis^7^ because it is fast, relatively non-invasive and readily available. Conversely, MRI has better soft tissue characterization than CT,^8^ but has some limitations such as longer acquisition times and lack of prompt availability on an emergency basis in several institutions, which further limits its potential role in the acute setting.

## CLINICAL PRESENTATION

A 29-year-old Caucasian female presented to the emergency department complaining persistent pain in the left iliac fossa of 3 days duration, soprapubic pain during micturition, nausea and vomiting. Her past medical history included splenectomy for hereditary spherocytosis at the age of 6 and cholecystectomy at the age of 22. On examination, her vital signs were normal. Physical examination revealed direct and rebound tenderness in the pelvis as well as abdominal distension.

## INVESTIGATIONS/IMAGING FINDINGS

Laboratory tests showed metabolic acidosis, elevated serum lactate and amylase, neutrophilic leukocytosis (13.6 u l^−1^), increased C-reactive protein (CRP; 10.6 mg dl^−1^, n.v. ≤1), and increased platelets count (409 × 10^9^ l^–1^, n.v. 130–400); renal function and serum beta human chorionic gonadotropin (hCG) levels were within the normal range. Gynaecological and surgical referral was advised.

**Figure 1.**
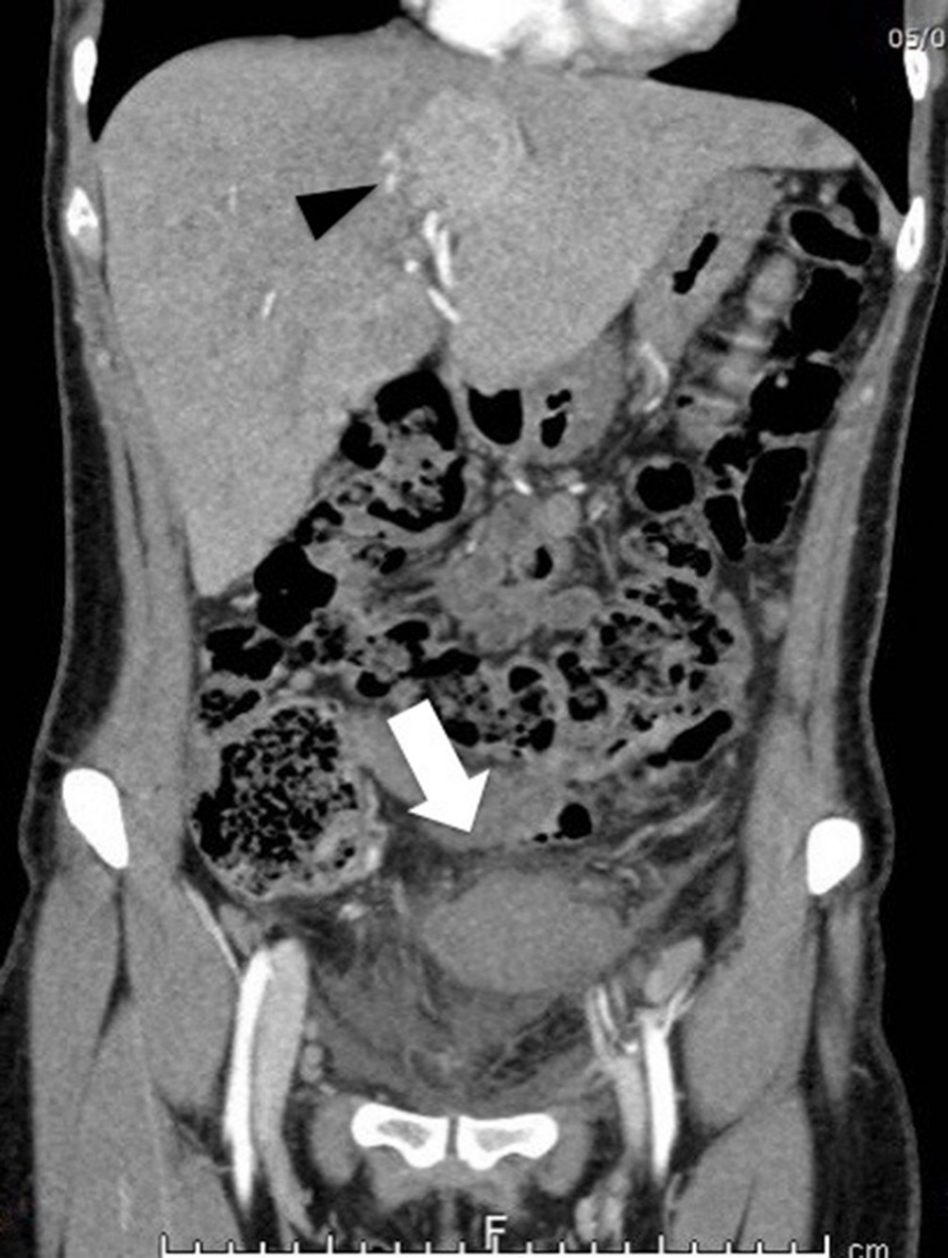
4 mm slab thickness maximum intensity projection (MIP) coronal contrast-enhanced imaging shows several lesions of variables size with different pattern of contrast-enhancement (arrowhead, arrow) in keeping with splenic implants.

**Figure 2.**
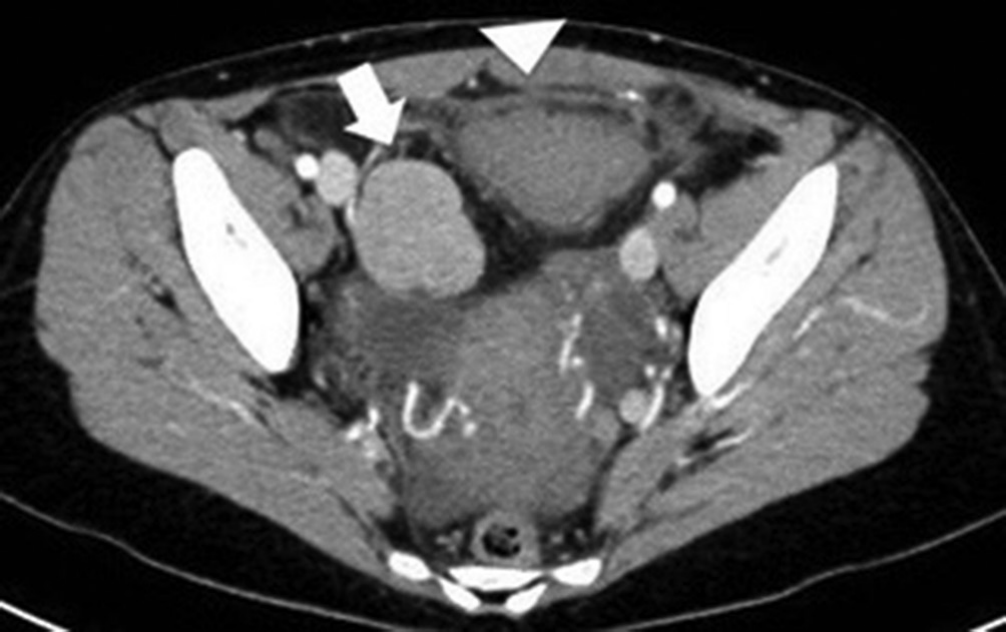
Axial contrast-enhanced CT scan shows poor contrast enhancement of the left splenic implant surrounded by fat stranding and a small amount of fluid in the recto-uterine pouch, in keeping with torsion (arrow head). There is a right contrast enhancing pelvic implant as well (arrow).

**Figure 3.**
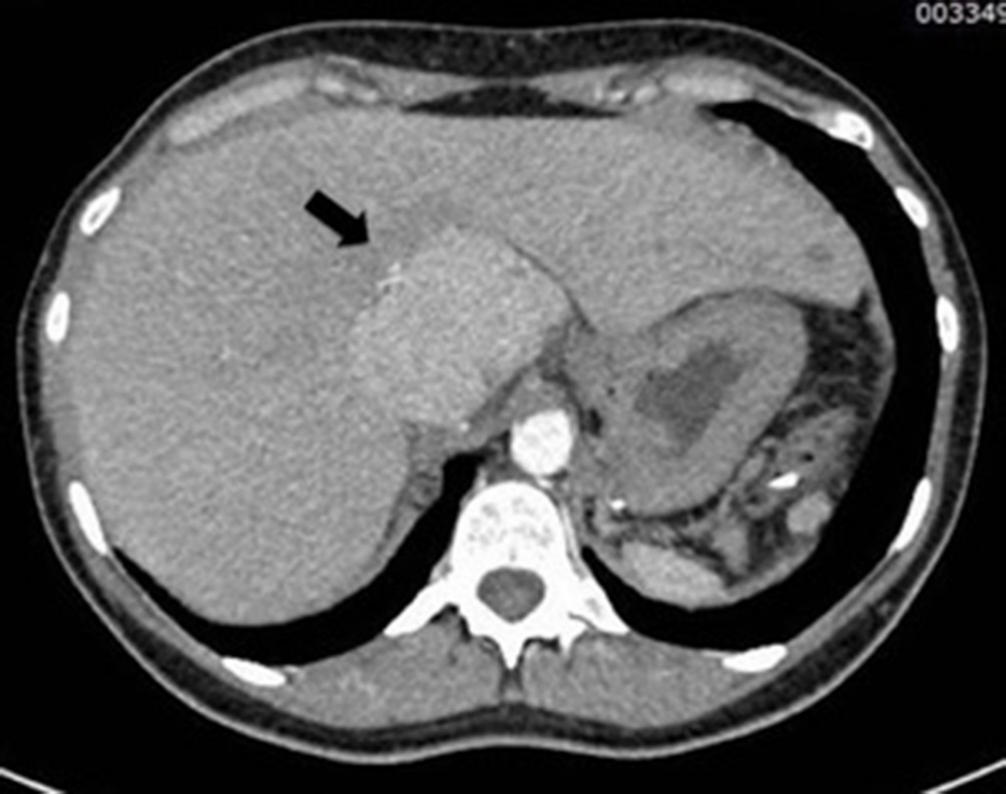
Axial CT scan shows a further contrast enhancing lesion in the liver in keeping with hepatic splenosis (arrow).

Subsequent plain abdominal X-rays (not showed) demonstrated no evidence of bowel obstruction or perforation. Dual phase arterial and portal venous CT scan of the abdomen and pelvis (SOMATOM Definition Flash, Siemens Healthcare, Forchheim, Germany), showed several contrast-enhancing masses up to 5 cm in transverse diameter (Figure 1), a mass adjacent to the urinary bladder with poor contrast enhancement surrounded by fat stranding (Figure 2) and a small amount of fluid in the recto-uterine pouch. Finally, an additional contrast-enhancing mass located at segment 1 of the liver was shown (Figure 3). The remaining CT findings were unremarkable. Two boluses of 5 ml each of indocyanine green (ICG)-enhanced fluorescence agent were administered intravenously during the laparoscopic procedure, which confirmed active vascular supply of the right pelvic splenic implant and poor/absent vascular supply of the left pelvic implant, in keeping with ischemia.

## DIFFERENTIAL DIAGNOSIS

According to the clinical presentation and imaging findings, the differential diagnosis includes endometriosis, torsion of a pedunculated fibroid and ovarian torsion. Owing to the past medical history of splenectomy for hereditary spherocytosis, splenosis should be also taking into account.

## TREATMENT

**Figure 4.**
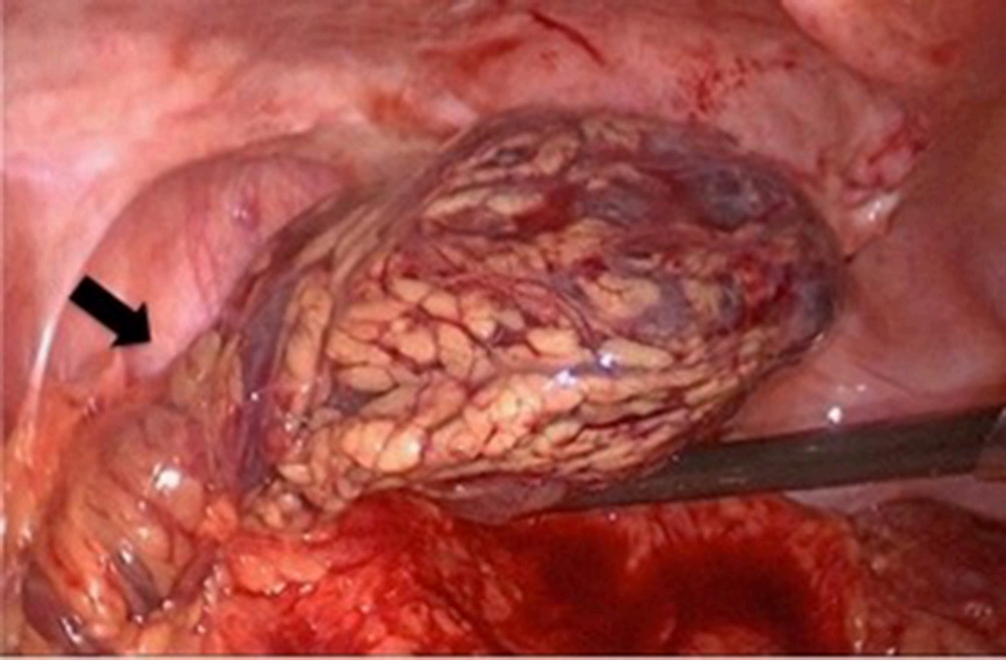
Surgical specimen shows a twisted vascular pedicle (arrow) with venous engorgement and ischemia.

The ischemic splenic implant was removed by laparoscopic surgery. The remaining lesions were not removed because they were not ischemic. Surgical specimen showed a twisted vascular pedicle with venous engorgement and ischemia (Figure 4). Pathologic examination of the resected specimen revealed vascular congestion of the red pulp and hypoplasia of the white pulp.

## OUTCOME AND FOLLOW-UP

The post-operative course was uneventful, and the patient was discharged from the hospital in good condition at day 8. She was followed up as outpatient conjointly by general practitioners and ambulatory surgeons. During follow-up (3 months), she remained asymptomatic.

## DISCUSSION

This case of torsion of a splenic implant in a context of abdominopelvic splenosis in a young adult female patient was managed as an acute abdomen. Clinically, the patient’s symptoms were highly suspicious for an acute bowel ischemia. Given this clinical scenario, rapid evaluation was necessary to identify any intra abdominal pathology requiring timely surgery. Thus, contrast-enhanced dual phase CT was the first-line imaging modality in our case.

Appearance of the splenic implants at ultrasound is non-specific. However, similar to multiplanar reformatted (MPR) images, color-Doppler ultrasound may be useful to identify the twisted pedicle when it is not extremely thin.^1^ Torsion of pedunculated fibroids is a rare complication, which is difficult to diagnose pre-operatively because the twisted pedicle is often extremely thin and the absence of contrast enhancement is not pathognomonic for ischemia. In our case, the presence of multiple masses led to rule out this condition. Similarly, adnexal torsion is commonly unilateral, with a slight (3:2) right-sided predilection.^9^ CT has lesser specificity for the diagnosis of endometriomas than MRI and thus plays a limited role in the evaluation of endometriosis. However, ovarian endometriomas may be cystic and often contain blood, whereas the appearance of the endometrial implants is generally non-specific.^9^ At contrast-enhanced CT, splenosis has comparable attenuation to that of normal splenic tissue (Figures 1–2). The intensity and enhancement of the splenic nodules on MRI are also similar to that of normal spleen. *T*_2_ and *T*_1_ weighted fat-suppressed MRI sequences play a critical role in the detection of the infarcted splenosis, showing hypointense signal on both *T*_1_ and *T*_2_ weighted sequences and no post-contrast enhancement on *T*_1_ weighted sequences.^7^

On unenhanced MRI weigthed sequences, endometriomas show very high signal intensity on *T*_1_, shading on *T*_2_, and poor to absent enhancement on post-contrast *T*_1_.^7^

Although MRI using superparamagnetic iron oxide (SPIO) is specific for the diagnosis of splenosis, the current diagnostic tool of choice is scintigraphy with reticuloendothelial agents such as ^99m^Tc sulphur colloid, ^99m^Tc heat-damaged erythrocytes or ^111^In-labeled platelets.^1^

The use of ICG-enhanced fluorescence agent during laparoscopy plays a confirmatory role in the diagnosis of perfusion abnormalities of solid and hollow viscous organs, and as in our case, to confirm an ischemic process of the splenic implant.^10^

## Conclusion

Splenosis torsion is extremely rare and it is still a diagnostic dilemma; the complication of abdominal splenosis should be considered in the differential diagnosis in patients with previous splenectomy. MPR images may assist in the identification of the vascular pedicle. Finally, comparison with previous imaging studies is helpful and should be sought whenever available.

## LEARNING POINTS

1. CT is useful in the emergency setting in the detection of splenosis.
2. *T*_2_ and *T*_1_ weighted fat-suppressed MRI sequences as well as post-contrast *T*_1_ weighted sequences play a critical role in the detection of splenosis infarction.
3. Appearance of the splenic implants at ultrasound is non-specific. However, color-Doppler ultrasound may be useful to identify any twisted pedicle.
4. ICG-enhanced fluorescence agent during laparoscopy plays a confirmatory role in the diagnosis of splenosis ischemia.
